# Uncovering syntrophic potential from genome-resolved metagenomics of suspended and granular anaerobic digestion sludges

**DOI:** 10.1093/femsec/fiag052

**Published:** 2026-05-21

**Authors:** Maaike S Besteman, Emilie Alaux, Anna Doloman, Guillaume Tahon, Thijs J G Ettema, Diana Z Sousa

**Affiliations:** Laboratory of Microbiology, Wageningen University & Research, Stippeneng 4, 6708 WE Wageningen, The Netherlands; Laboratory of Microbiology, Wageningen University & Research, Stippeneng 4, 6708 WE Wageningen, The Netherlands; Laboratory of Microbiology, Wageningen University & Research, Stippeneng 4, 6708 WE Wageningen, The Netherlands; Laboratory of Microbiology, Wageningen University & Research, Stippeneng 4, 6708 WE Wageningen, The Netherlands; Laboratory of Microbiology, Wageningen University & Research, Stippeneng 4, 6708 WE Wageningen, The Netherlands; Laboratory of Microbiology, Wageningen University & Research, Stippeneng 4, 6708 WE Wageningen, The Netherlands

**Keywords:** metagenomics, syntrophy, anaerobic digestion, fatty acid oxidation

## Abstract

Syntrophic microbial interactions are fundamental to the degradation of organic matter (e.g. fatty acids), playing a central role in natural anoxic ecosystems and engineered systems such as anaerobic digestion (AD). Despite their ecological and biotechnological importance, only a limited number of (obligate) syntrophic fatty-acid oxidizers have been successfully isolated. In this study, microbial communities from suspended and granular sludge samples were characterized using 16S rRNA gene amplicon sequencing and shotgun metagenomics. Network analysis of the 16S rRNA gene amplicon data revealed strong positive associations between methanogens and known syntrophic fatty-acid oxidizers, particularly in granular sludge samples. 743 High-Completion Metagenome Assembled Genomes (HC-MAGs) were recovered. This comprehensive HC-MAGs dataset provides a valuable resource for identifying novel microorganisms with genomic potential for syntrophic oxidation of butyrate, propionate, and acetate. This analysis identified multiple interesting novel targets, including the families DTU052 and CALXsZ01 (class Syntrophomonadia) as potential butyrate oxidizers; the families UBA6807, PHBD01, FEN-1087, and FEN-1099 (class Syntrophia) as potential propionate oxidizers; and genus DTU068 (family Thermacetogeniaceae) together with the family-level lineage 4572–78 (phylum Chloroflexota) as potential acetate oxidizers. These findings highlight granular sludges as a reservoir for previously uncharacterized syntrophic microorganisms. The recovered HC-MAG dataset also provides a framework to further elucidating fatty-acid oxidizing bacterial lineages within complex anaerobic communities.

## Teaser

Metagenomic investigation of suspended and granular anaerobic digestion sludges suggests novel syntrophic potentials.

## Introduction

In anoxic environments, microorganisms form complex communities characterized by intricate trophic networks, where cooperation is driven by the limited availability of substrates (i.e. carbon sources, electron donors) and suitable electron acceptors. When CO_2_ serves as the primary or sole electron acceptor, the degradation of certain compounds, such as fatty-acids and alcohols, requires the cooperation between the organism oxidizing these substrates and hydrogen/formate-utilizing methanogens. By maintaining low hydrogen partial pressure and/or formate concentration, methanogens render these conversions thermodynamically favorable. This tightly coupled, thermodynamically constrained interaction is known as syntrophy (McInerney et al. 2008, Narihiro et al. 2015, Yue et al. 2021). Syntrophy plays a vital role in both natural environments, such as sediments or wetlands, and engineered anoxic ecosystems, including anaerobic digesters.

In anaerobic digestion (AD), a complex microbial community converts organic compounds into biogas through a stepwise process involving hydrolysis, acidogenesis, acetogenesis, and methanogenesis (Yadav et al. 2022). During the initial steps, intermediates such as alcohols, amino-acids, aromatic compounds, long-chain fatty acids (LCFAs), and volatile fatty acids (VFAs) are formed. Their subsequent conversion relies on syntrophic interactions between specific bacteria (i.e. syntrophic bacteria) and methanogens—processes often considered rate-limiting in AD (Jin and Lu 2023, Wu et al. 2022, Zhang et al. 2018). The AD process is widely used for treating domestic and industrial wastewaters, sewage sludges, and other organic wastes. The final product, biogas, is an energy carrier that can be used for electricity production or upgraded to biomethane and injected into the gas grid (Scarlat et al. 2018). The digestate from AD can be used as organic sustainable fertilizer on agricultural lands, increasing for example grass yield and biodiversity and improving soil health (Brychkova et al. 2024, Scarlat et al. 2018).

The total number of characterized syntrophs remains low, with isolates belonging exclusively to the phyla Bacillota (previously Firmicutes) and Desulfobacterota. For example, only 12 syntrophic butyrate oxidizing bacteria (SBOB) have been isolated to date, mostly belonging to the family Syntrophomonadaceae and a single representative to Syntrophaceae (Müller et al. 2010, Sousa et al. 2009, Ziels et al. 2019). Some of these bacteria also oxidize LCFAs and medium-chain fatty acids. *Syntrophomonas wolfei* was the first isolated fatty-acid oxidizing syntroph (capable of oxidizing C4 to C8 fatty-acids), and has since served as model organism in syntrophy research (McInerney et al. 1979, 1981, Schmidt et al. 2013). Syntrophic propionate oxidizing bacteria (SPOB) include *Syntrophobacter fumaroxidans* MPOB (Harmsen et al. 1998), *Smithella propionica* LYP^T^ (Liu et al. 1999), *Pelotomaculum propionicum* MGP^T^ (Imachi et al. 2007), *Pelotomaculum thermopropionicum* SI^T^ (Imachi et al. 2002). Syntrophic acetate oxidizing bacteria (SAOB) isolates are limited to only three with a known SAO pathway identified: *Syntrophaceticus schinkii* Sp3^T^ (Manzoor et al. 2016, Westerholm et al. 2010), *Thermacetogenium phaeum* PB^T^ (Hattori et al. 2000, Oehler et al. 2012), and *Tepidanaerobacter acetatoxydans* Re1^T^ (Westerholm et al. 2011). Several of these syntrophs originate from AD sludges, despite typically being present in low abundance in AD systems (Stams et al. 2012). While the low abundance of known syntrophs does not necessarily reflect a limited ecological role, it is possible that other, yet uncharacterized lineages contribute to similar functions as syntrophic VFA oxidizers. Given that AD sludges have already been shown to be a good source for syntroph enrichments, they remain an ideal starting point for the identification and isolation of novel syntrophic candidates.

Syntrophic fatty-acid oxidizers utilize different pathways depending on the substrates they use. Butyrate and longer fatty acids are primarily oxidized via the β-oxidation pathway (Sousa et al. 2009). SPOB, such as *P. propionicum* and *S. fumaroxidans*, employ the methylmalonyl-CoA (MMC) pathway, although the exact mechanism for propionate activation is not known (Hidalgo‐Ahumada et al. 2018). Additionally, a second propionate oxidation pathway is proposed for *S. propionica*, involving dismutation of 2 mol of propionate into butyrate and acetate (De Bok et al. 2001, Liu et al. 1999). Yet, the enzymes involved in this pathway are largely unknown. In SAOB, acetate is oxidized via the reverse Wood-Ljungdahl Pathway (WLP), also referred to as the reverse acetyl-CoA pathway. This pathway has been confirmed in *S. schinkii* Sp3^T^ (Manzoor et al. 2016, Westerholm et al. 2010), *T. phaeum* PB^T^ (Hattori et al. 2000, Oehler et al. 2012), and members of *T. acetatoxydans* (Westerholm et al. 2011). For the SAOB *Schnuerera ultunensis* (formerly *Clostridium ultunense*) and *Pseudothermotoga lettingae*, a variant of the reversed WLP, the glycine cleavage pathway, has been postulated (Nobu et al. 2015, Schnürer et al. 1996, Zeng et al. 2021). However, the genetic basis and functional activity of this pathway in SAO have not been experimentally confirmed.

In the present study, we have made an effort to uncover potential novel SBOB, SPOB, and SAOB, in 117 suspended and granular AD sludges collected from various digesters across the Netherlands. First, the microbial diversity in AD sludges was assessed by 16S rRNA gene amplicon sequencing. Second, in-depth metagenomic analyses were conducted on a selection of 14 AD sludges, enabling the identification of both known and potentially novel syntrophs through the screening of key genes involved in butyrate, propionate and acetate oxidation pathways. As an output, we gained insight into the abundance and distribution of both known and potential novel syntrophs in granular versus suspended AD sludges. Importantly, the identification of novel candidates provides a basis for targeted cultivation, where their isolation and study on their physiology can further improve our understanding of the microorganisms supporting efficient biogas production in AD systems.

## Methods

### AD sample collection

A total of 117 anaerobic sludge samples were collected from anaerobic digesters across The Netherlands (File S1, “AD_metadata”; Table 1). Of each AD system, information on bioreactor type, feedstock, operation temperature, and pH were collected at the time of sampling. Of the collected samples, 106 were suspended sludges, while 11 were granular. Most of the suspended sludges (102 samples) came from AD systems processing sewage sludge waste from municipal waste-water treatment facilities; 4 sludge samples were derived from decomposing livestock agricultural waste. The granular sludges came from reactors fed with wastewater from the potato industry, tanneries, or recycled paper industry. From all sampled AD systems, up to 800 ml of sludge was collected, and immediately sub-sampled and stored at −20°C for further processing for 16S rRNA gene and metagenomic sequencing.

**Table 1:** Origin and characteristics of AD sludge samples used for 16S rRNA gene and metagenomic sequencing.

Reactor type	Temperature	Feedstock	Sludge type	#Samples 16S rRNA gene sequencing	#Samples metagenomic sequencing
IC	30–44°C	Municipal/industrial WWTP sludge	Suspended	96	6
IC	49–56°C	Municipal/industrial WWTP sludge	Suspended	6	1
IC	40–43°C	Agricultural waste (including livestock manure)	Suspended	4	2
UASB	32–37°C	Industrial water of potato industry	Granular	9	3
IC	31°C	Tannery wastewater, high salinity	Granular	1	1
EGSB	36°C	Recycled paper	Granular	1	1

Abbreviations: IC, Internal Circulation; UASB, Upflow Anaerobic Sludge Bed; EGSB, Expanded Granular Sludge Bed; WWTP, wastewater treatment plant.

### 16S rRNA gene community profiling and network association analysis

DNA was extracted from 400 µl of each sludge with the DNeasy PowerLyzer PowerSoil Kit (Qiagen™, Venlo, The Netherlands) according to supplier’s instruction, except that cell lysis was promoted in three consecutive cycles of 45 s bead beating at 4 m/s, with 25 s resting steps in between. DNA was quantified using the Invitrogen™ Qubit dsRNA BR assay kit (Thermo Scientific™, Waltham, MA, USA) and stored at −20°C until further processing. The 16S rRNA gene libraries were prepared using universal primers developed within the Earth Microbiome Project (EMP-F: 5′-GTGYCAGCMGCCGCGGTAA-3′; EMP-R: 5′-GGACTACNVGGGTWTCTAAT-3′) (Caporaso et al. 2011, Gilbert et al. 2014). The resulting PCR products were indexed using 10 nt indices and pooled equimolarly to create an amplicon library. The amplicon pool was run at 70 V for 2 h on a 1% agarose, cut-out and purified using the GeneJET PCR Purification Kit (Thermo Scientific™, Waltham, MA, USA), to remove primer dimers and non-specific amplicons. Subsequently, the DNA in the library was quantified using Qubit dsDNA quantification kit (Thermo Scientific™, Waltham, MA, USA) and subsequently sequenced and demultiplexed with an in-house Illumina iSeq100, yielding 2 Gb of raw data. PhiX was spiked at 25%.

Forward and reverse reads of the amplicons did not overlap, therefore only forward reads were used for analysis, as they provide greater taxonomic resolution than reverse reads. First, the EMP-F primer and poly G tails of at least 15 nucleotides long were removed from all reads using Cutadapt v2.9 (Martin 2011). Reads shorter than 120 bp were discarded. Subsequent analysis was conducted using QIIME 2 v2020.2 (Bolyen et al. 2019). Within QIIME 2, reads were quality-filtered with a minimum PHRED score threshold of 30 for every nucleotide, denoised using DADA2 with the consensus chimera method, and clustered via an open-reference method at 97% similarity. Taxonomic classification was performed using an in-house optimized version of the SILVA 138.1 SSURef_NR99 database (Quast et al. 2013), constructed through the RESCRIPt pipeline and augmented with additional 16S rRNA gene sequences from recently discovered archaeal taxa (e.g. Asgardarchaeota, Bathyarchaeota, Korarchaeota). Further statistical analysis and data visualization were carried out on R CRAN v4.3.2 (R core team 2018), managing data with the phyloseq package (McMurdie and Holmes 2013). Out of the 117 AD sludge samples, 3 were removed due to insufficient sequencing depth (<350 reads per sample). The total sum scaling method was used to express relative abundances (RA) of taxa. To compare the microbial communities of granular and suspended sludges, a subset was extracted from the overall dataset: the granular sludge group consisted of 8 samples, while suspended sludge samples were selected only if they matched the pH and temperature ranges of the granular samples. Specifically, the temperature range was set between 33.0 and 37.0°C, and the pH range between 7.0 and 7.3. This selection process resulted in a total of 17 suspended sludge samples and 8 granular sludge samples. Microbial association networks in the selected suspended and granular sludge samples were inferred using the SPRING method (Yoon et al. 2019) as the association measure within the NetCoMi package (Peschel et al. 2021). For the analysis, only the 100 most frequently occurring taxa were retained. Network clusters were identified using greedy modularity optimization. Bacterial taxa recognized as obligate syntrophs in the literature (File S1, “Syntrophs”) were categorized as “known syntrophs” and highlighted in the analysis of the selected subset of samples.

### Metagenomic analysis

Based on the amplicon sequencing results, a total of 14 samples was selected for full metagenome sequencing (File S1, “AD_metadata”; Table 1). For that, DNA was extracted from 400 µl of each sludge with the DNeasy PowerLyzer PowerSoil Kit (Qiagen™, Venlo, The Netherlands). DNA was sequenced by Illumina whole-genome sequencing (WGS) using the Novoseq 6000 platform (Novogene™, Cambridge, UK) with a prokaryotic library yielding paired-end reads of 150 bp. For each sample, 25 Gb of data was collected for subsequent metagenomic assembly.

Raw reads were cleaned by removing sequencing adapters, reads with PHRED quality score ≤ 5 and reads with more than 10% unidentified bases. Clean reads from each sample were individually assembled with MegaHit (v1.2.9) (Li et al. 2016). Binning was conducted by metaWRAP (v1.3.2) (Uritskiy et al. 2018), combining binning algorithms concoct 1.0 (v1.0.0), maxbin 2.0 (v2.2.6), and MetaBAT 2 (v2.12.1) (Alneberg et al. 2013, Kang et al. 2019; Wu et al. 2016). Final bins were constructed using the bin-refinement module of MetaWRAP on the three bin-sets, including CheckM (v1.0.12) for quality and completeness of the bins (Parks et al. 2015). Bins with completeness <50% and contamination >10% were excluded from bin refinement. For further analysis, only MAGs of High-Completion were included, considering the minimum information about a metagenome assembled genome (MIMAG) standard of CheckM (completeness >90% and CheckM contamination <5%) (Bowers et al. 2017). In addition to use of CheckM1, MAG quality was also analyzed with CheckM2 (v1.0.2) (Chklovski et al. 2023). From CheckM2 analysis, MAGs with <80% completion or >5% contamination were excluded from subsequent analyses. The remaining MAGs are referred to as High-Completion Metagenome Assembled Genomes (HC-MAGs). The selected MAGs were functionally annotated with the Annotate_bins module of Metawrap (Prodigal v2.6.3 and HMMER v3.4) and abundances were quantified with the quant_bins module of Metawrap (v1.3.2). The Classify_wf module of GTDB-Tk (v2.3.2) was used for Taxonomic classification of all MAGs, using reference data version R214 (Chaumeil et al. 2020, 2022). All 16S, 23S, and 5S rRNA gene sequences were identified using barrnap v0.9 (Torsten Seemann 2018). Of the HC-MAGs with more than one 16S rRNA gene sequences identified, taxonomic classification of all 16S rRNA gene sequences was checked by NCBI BLAST (Johnson et al. 2008, McGinnis and Madden 2004) to compare with the full MAG’s taxonomic classification of GTDB-Tk. Contigs containing a 16S rRNA gene classified different from the GTDB-Tk marker classification were removed. If multiple different 16S rRNA genes were identified with all different taxonomic classifications, not matching the GTDB-Tk marker classification, the complete MAG was excluded from further analysis.

### Syntrophic VFA oxidation pathway selection

The translated coding sequences of the HC-MAGs were used to generate a BLAST database with the makeblastdb tool from BLAST+ version 2.15.0 (Altschul et al. 1990, Kumar 2019). Known metabolic pathways for the oxidation of butyrate, propionate, and acetate were selected for targeted searches within the created metagenomic database. To identify MAGs with the potential of syntrophic butyrate oxidation, proteins involved in the butyrate β-oxidation pathway were obtained from *S. wolfei* strain Goettingen G311 (GenBank CP000448.1). For propionate oxidation, proteins involved in the MMC pathway were obtained from *S. fumaroxidans* strain MPOB (GenBank CP000478.1) (Jin and Lu 2023). And, for acetate oxidation, proteins involved in the reversed WLP pathway were obtained from *S. schinkii* (GenBank CDRZ01000009.1) (Manzoor et al. 2016). Homologous proteins in the HC-MAGs were identified using Basic Local Alignment Search Tool (BLAST) application BlastP with a minimum query coverage of 25 and E-value threshold 0.01 (Altschul et al. 1990, 1997). To determine which MAGs could be considered potential candidates for syntrophic oxidation of butyrate, propionate or acetate, pathway-specific selection criteria were applied:

- MAGs were considered to have the potential for butyrate oxidation if they encoded homologs with >40% sequence identity for at least five out of seven steps of the butyrate β-oxidation pathway.
- MAGs were considered to have the potential for propionate oxidation if they encoded at least one homolog for each of the steps in the MMC pathway and at least three homologs for the propionate activation step.
- MAGs were considered to have the potential for acetate oxidation if they encoded four enzymes of the reversed WLP: acetate kinase (Ak), methylene tetrahydrofolate (THF) dehydrogenase, methenyl THF cyclohydrolase, and formyl THF synthetase.

A total of 61, 63, and 98 MAGs meeting the criteria for butyrate, propionate, and acetate oxidation potential, respectively, were identified and placed in a phylogenetic tree with iq-Tree (see subsection “Phylogenetic trees”). Of the MAGs clustering as identical, sharing the same GTDB-Tk classification and having a similar protein homolog composition, only the most complete MAG was included in visualization of the homologs for the pathway. Homolog data of all MAGs are included in File S1 (tabs “Butyrate,” “Propionate,” “Acetate”).

For all MAGs selected with the genomic potential for butyrate, propionate, or acetate oxidation, the presence of six protein domains predicted to be characteristic for syntrophs was checked (Worm et al. 2014). Selected protein domains were IPR006443 of extra-cytoplasmic formate dehydrogenase FDH, IPR024064 and IPR006452 of the FDH maturation protein FdhE, IPR019079 of capsule synthesis protein CapA, IPR018365 of cell cycle proteins FtsW, RodA, and SpoVE, and IPR020539 of a conserved site of Ribonuclease P (RNaseP). Due to the exploratory nature of their association with syntrophy, these protein domains are hereafter referred to as Syntrophic Growth-Associated (SGA) Protein Domains. A higher weight was given to the presence of the extra-cytoplasmic Fdh and its maturation protein FdhE, due to their established role in formate interspecies electron transfer; the functions of the capsule synthesis, cell-cycle and Ribonuclease P in syntrophy remain elusive, making them less suitable as marker proteins. The protein sequences including the SGA protein domains of *S. wolfei, S. fumaroxidans, S. schinkii*, and *T. phaeum* were selected from available sequences on the InterPro database (Blum et al. 2021). Homologous proteins in the MAG-database were subsequently identified using BlastP with a minimum query coverage of 25 and E-value threshold 0.01.

### Phylogenetic trees

Phylogenetic trees were reconstructed for (1) all bacterial high completion MAGs and (2) for all MAGs selected for fatty-acid oxidation pathway visualization. For MAGs selected for phylogenetic placement, 120 bacterial marker genes were identified and aligned using GTDB-Tk v2.3.2. Alignments were trimmed with trimal v1.4.rev15 (-gappyout). For tree reconstruction, the best-fit substitution model using the -m MFP modelfinder parameter of iq-Tree v2.3.4 (Nguyen et al. 2015). Final trees were reconstructed using 1000 standard non-parametric fast bootstrap replicates. Tree visualization was performed in Itol v6.9.1.

### Data

Raw sequencing data and the assembled HC-MAGs were deposited to the European Nucleotide Archive at project PRJEB87655, with study accession ERA31233818.

## Results

**Figure 1:**
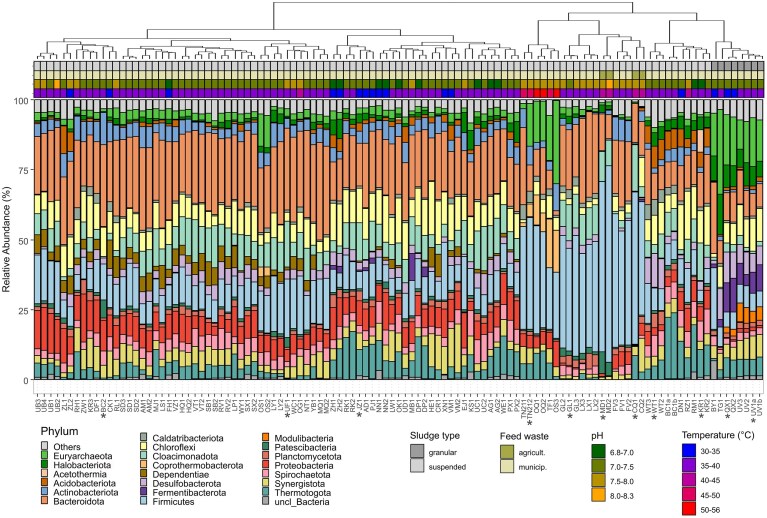
Overview of the microbial community in 111 WWTP anaerobic digesters, based on 16S rRNA gene amplicon sequencing classified with SILVA (v138.1). Bar chart representing the microbial relative abundances displayed at the taxonomic Phylum level. Phyla with a relative abundance representing <5% in all samples are gathered as “Others.” Samples are clustered according to a weighted Unifrac distance on the *x*-axis. Metadata (sludge type, feed waste, pH, and temperature) collected during the sampling of the different digesters are indicated on the top of the bar chart. Stars indicate the eleven samples selected for full metagenome sequencing.

A comprehensive 16S rRNA gene based microbial diversity analysis was conducted on AD sludge samples collected across The Netherlands (File S1, “AD_metadata”—“Sequencing Run 1”; 111 samples with high-quality sequencing data). Between 13 000 and 113 000 high-quality sequences per sample were obtained, yielding a total of 5351 non-redundant operational taxonomic units (OTUs). The taxonomic affiliation of these OTUs at the phylum level resulted in 72 distinct phyla (SILVA 138.1), of which 50 individually accounted for <5% of the abundance in all samples. These minor phyla were gathered as “Others”; the remaining 22 phyla account for 95.8 ± 1.7% of the total diversity (Fig. 1).The analysis of the microbial communities first revealed that generally digesters from the same WWTP cluster together. In addition, four primary clusters were identified and linked to sampling metadata, underscoring associations between microbial composition, feed type, and operational conditions, from left to right

- The largest cluster, comprising 84 samples, was predominantly fed with municipal wastewater sludge, exhibiting a predominance of Bacteroidota [relative abundance (RA) 22%], Firmicutes (RA 11%, GTDB classification Bacillota), and Chloroflexi (RA 10%, GTDB classification Chloroflexota), with a relatively low representation of archaea (RA 4%)
- A cluster consisting of 8 samples derived from granular sludge showed a high abundance of Euryarchaeota (RA 17.3 ± 5.4%, GTDB classification Thermoplasmatota and Methanobacteriota) and Halobacteriota (RA 8.2 ± 2.6%), resulting in a total RA of archaea to 25.5%. In terms of bacterial composition, this cluster mainly comprises members of Bacteroidota (RA 11.5 ± 5.4%), Chloroflexi (RA 8.5 ± 2.6%), and Firmicutes (RA 8.3 ± 5.2%).
- Six samples from digesters subjected to high temperatures, ranging from 49 to 56°C, displayed a high abundance of Firmicutes (RA 32.5 ± 7.2%), alongside variable RA of Bacteroidota (RA 11.9 ± 8.3%) and Euryarchaeota (RA 15.3 ± 8.1%). Notably, this cluster contained members of the Coprothermobacterota phylum (RA 9.4 ± 5.6%), which was not detected in any of the other samples.
- The final cluster comprised 13 samples, four of them sourced from WWTP that received agricultural waste. This group exhibited a high RA of Firmicutes (RA 46.4 ± 10.3%) and Bacteroidota (RA 20.3 ± 7.1%). This group also displayed a remarkable abundance of Cloacimonadota (RA 11.7 ± 2.1%) in 10 out of the 13 samples (excluding the FV WWTP).

**Figure 2:**
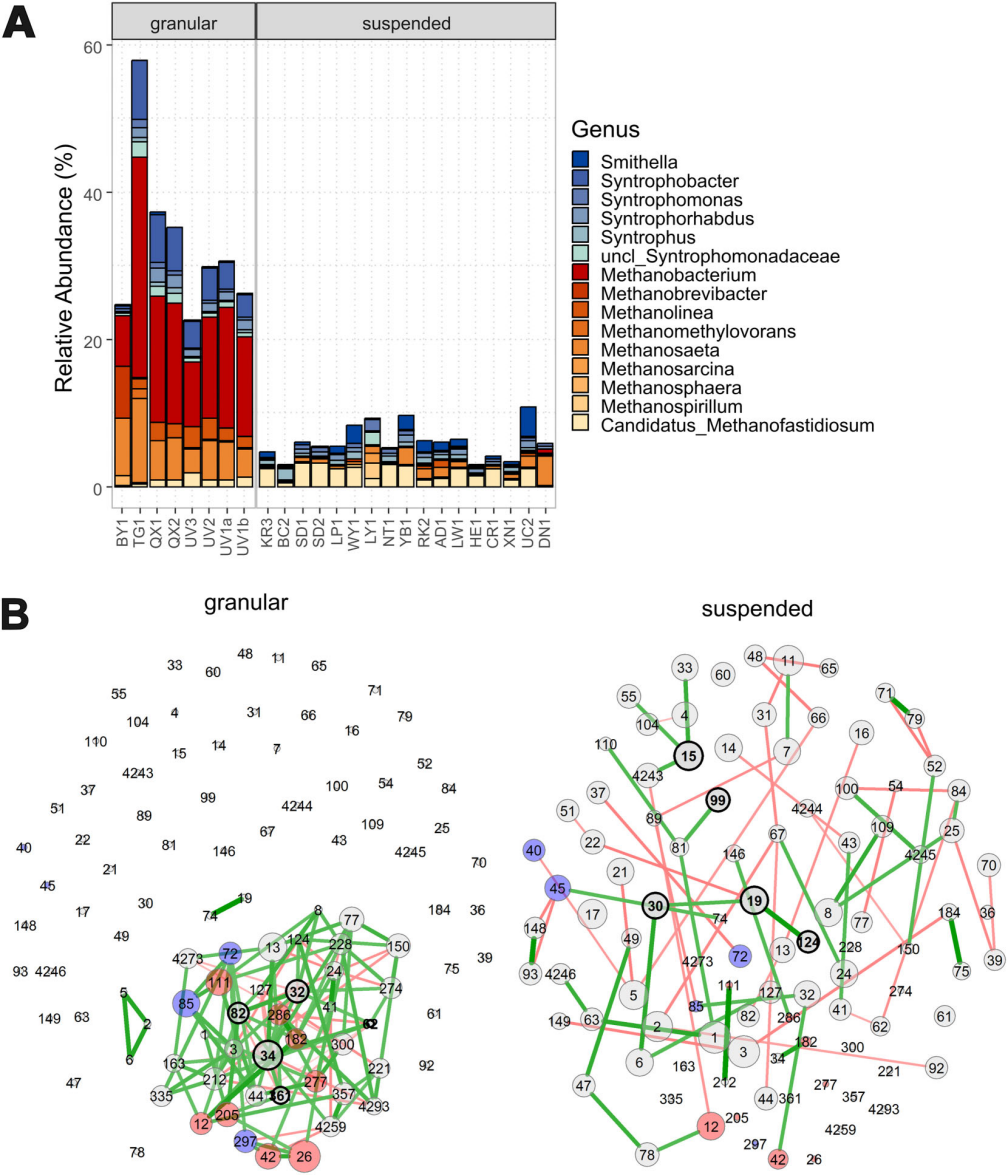
Comparison of the granular and suspended sludge communities, based on 16S rRNA gene amplicon sequencing classified with SILVA (v138.1). (A) Bar chart representing the microbial relative abundances of methanogens (red) and known syntrophs (blue), displayed at the taxonomic genus level, in granular and suspended sludges. (B) Microbial network associations for the combined dataset of granular and suspended sludges. Each number represents one OTU and a node of the network. Green edges correspond to positive estimated associations and red edges to negative ones; red nodes correspond to methanogen genera and blue nodes to currently known syntrophs; size of the node corresponds to CLR-normalized abundance of the corresponding OTU.

Overall, these results underscore the distinct microbial community structure associated with different feed sources and operational conditions in the sampled AD systems. To further investigate potentially new syntrophic interactions, particularly involving methanogenic archaea, the relative abundances of methanogens and known syntrophs were analyzed (Fig. 2A). Both groups exhibited higher RA in granular sludge samples—14.5 ± 12.2% for methanogens and 5.1 ± 3.7% for syntrophs—compared to the suspended sludges, which exhibited 3.6 ± 1.3% and 2.5 ± 1.3%, respectively. Based on this enrichment of methanogens and syntrophs in granular sludge, we subsequently focused our analysis on these samples. A co-occurrence of *Syntrophobacter, Methanobacterium*, and *Methanosaeta* was observed in 7 out of 8 granular sludge samples. Sample BY1 contained significantly fewer syntrophs and a noticeable presence of *Methanobrevibacter*.

A microbial network comparative analysis also revealed clear differences in the estimated associations between the two sludge types (Fig. 2B), with each group demonstrating unique clustering patterns and hub nodes. By examining the OTUs (i.e. nodes) and their interactions (i.e. edges) within the network, their contribution to microbial community assembly can be analyzed. Particularly, within the granular sludges, a cluster of strong positive associations was identified around a few nodes, with currently known syntrophs and methanogens (File S1, “Network”). The density of positive interactions between these two groups of interest in this cluster suggests that granular sludge should be included when investigating for potential novel syntrophs.

### MAGs recovered from granular and suspended AD sludges

**Figure 3:**
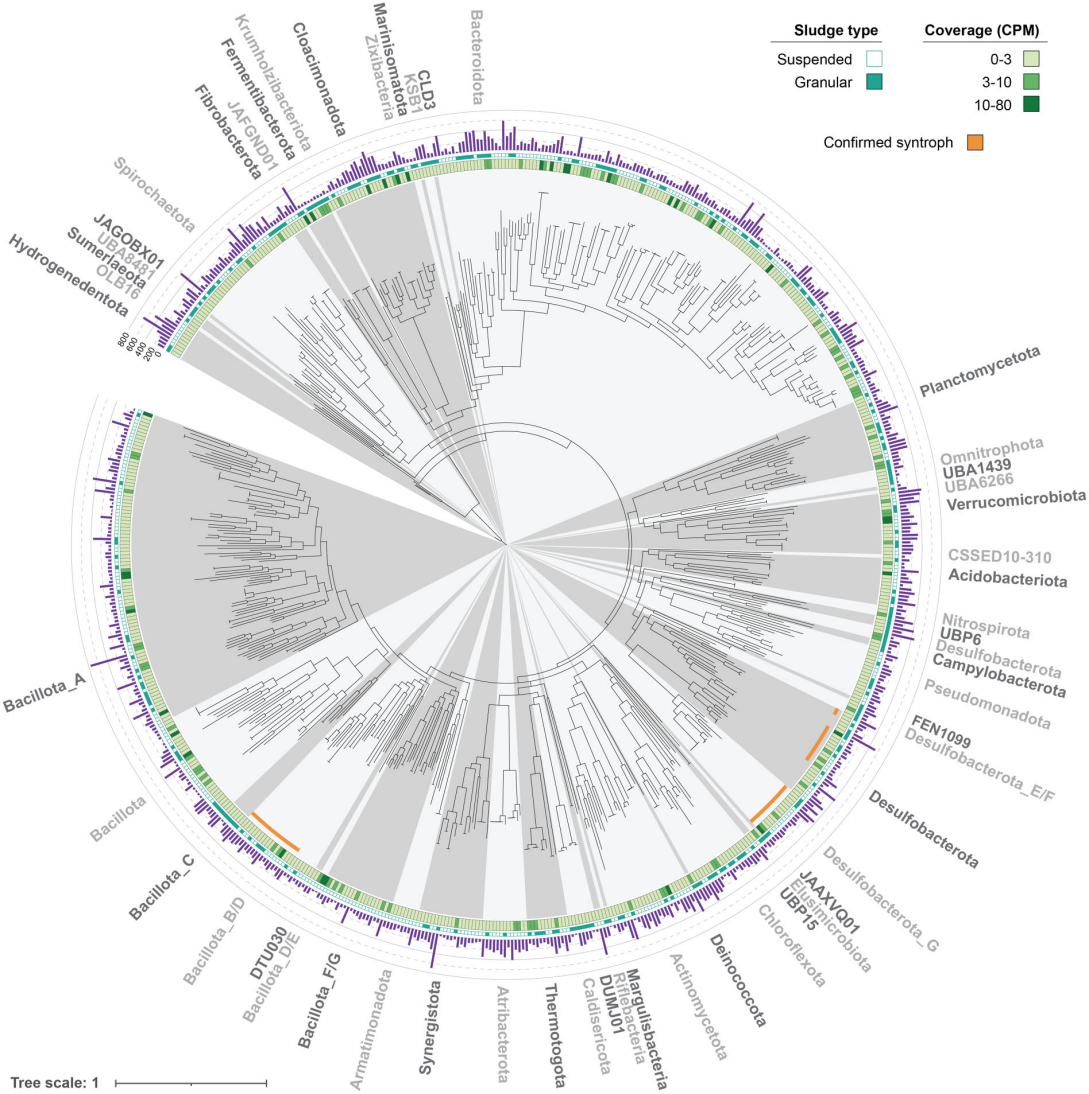
Phylogenetic tree of all High-Completion bacterial MAGs recovered in this study. The tree is based on a trimmed alignment of 120 bacterial marker genes using GTDB-Tk and is constructed with substitution model LG+R10 (Nuclear region with general matrix and FreeRate model) and 1000 non-parametric bootstrap replicates. The coverage in copies per million (CPM) of each MAG is indicated by heatmap. The purple bar chart represents the amount of contigs each MAG consists of.

Fourteen AD sludge samples were selected for WGS, including samples from all four primary clusters identified by the 16S rRNA gene diversity analysis (Fig. 1). This resulted in 743 High Completion (HC) metagenome assembled genomes (MAGs). The short-read sequences combined with the nature of highly conserved regions in the 16S rRNA gene hampered incorporation of this gene sequence during assembly and binning. MIMAG standards of presence of 23S, 16S, and 5S rRNA genes and at least 18 tRNA for high-quality genomes could therefore only be met for 157 MAGs (File S1, “MAGs”) (Bowers et al. 2017). The 743 MAGs are therefore further referred to as HC-MAGs. The 66 archaeal HC-MAGs were represented by 35 MAGs of the phylum Halobacterota, 21 MAGs of the phylum Methanobacteriota, 8 of the phylum Thermoplasmatota, and 2 of the phylum Thermoproteota (File S1, “MAGs”). With the focus on a search for novel syntrophic bacteria, the 677 bacterial HC-MAGs–428 retrieved from suspended AD sludge and 249 from granular AD sludge, respectively—were placed in a phylogenetic tree, thus showing the diversity of recovered phyla (Fig. 3). The phyla with the overall highest representation were found to be Bacillota (177 MAGs) and Bacteroidota (151 MAGs).

Although the majority of bacterial MAGs originated from suspended sludges (428 of 677), several phyla were predominantly recovered from granular sludge samples (Table 2). These included uncharacterized or poorly characterized phyla, including the phyla Chloroflexota, Caldisericota, Fermentibacterota, Bacillota_C (class Negativicutes), and Omnitrophota. A distinct cluster of four MAGs belonging to the phylum Desulfobacterota, was located in between the phyla UBP6 [proposed *Candidatus* Asafiota (Pallen et al. 2022)] and Campylobacterota. These Desulfobacterota MAGs originate all from granular sludge, as do the representatives of phyla *Ca*. Asafiota and Campylobacterota. Two of the MAGs belong to the family Desulfomicrobiaceae and the other two MAGs to the family Desulfovibrionaceae. The other 47 Desulfobacterota MAGs were placed in one clade together with one MAG of the phylum FEN-1099. This lineage, proposed *Candidatus* Lernaellota or *Candidatus* Podoxiota (Pallen et al. 2022, Williams et al. 2022), is most closely related to Desulfobacterota groups E and F that are in this study only recovered from granular sludge samples. Overall, these novel lineages found in granular sludges underline the microbial diversity within granular sludge communities which require more in-depth investigation.

**Table 2:** Phyla assigned to MAGs recovered from either granular or suspended sludge samples.

Granular sludge		Suspended sludge	
Chloroflexota (13/20) Caldisericota (8/9) Fermentibacterota (7/8) Bacillota_C (6/6) Omnitrophota (5/5) KSB1 (3/3) Nitrospirota (3/3) UBP6 (3/3) Campylobacterota (2/2) Desulfobacterota_E (2/2) Desulfobacterota_F (2/2) Zixibacteria (2/2)	OLB16 (1/1) CLD3 (1/1) Deinococcota (1/1) Elusimicrobiota (1/1) JAAXVQ01 (1/1) JAFGND01 (1/1) Krumholzibacteriota (1/1) Margulisbacteria (1/1) Riflebacteria (1/1) UBA6266 (1/1) UBA8481 (1/1)	Bacillota_G (20/20) Armatimonadota (8/8) Bacillota_D (5/5) Fibrobacterota (4/4) DTU030 (2/2) DUMJ01 (2/2) UBP15 (2/2) Bacillota_E (1/1) Bacillota_F (1/1) CSSED10-310 (1/1) FEN-1099 (1/1) JAGOBX01 (1/1)	Marinisomatota (1/1) UBA1439 (1/1)

Phyla with an outstanding representation compared to the total granular/suspended MAG recovery are included. For both types of sludge origin, the number of MAGs representing each phyla is presented in as (#_in sludge type/#_total).

### Exploring syntrophic butyrate oxidation in AD MAGs

**Figure 4:**
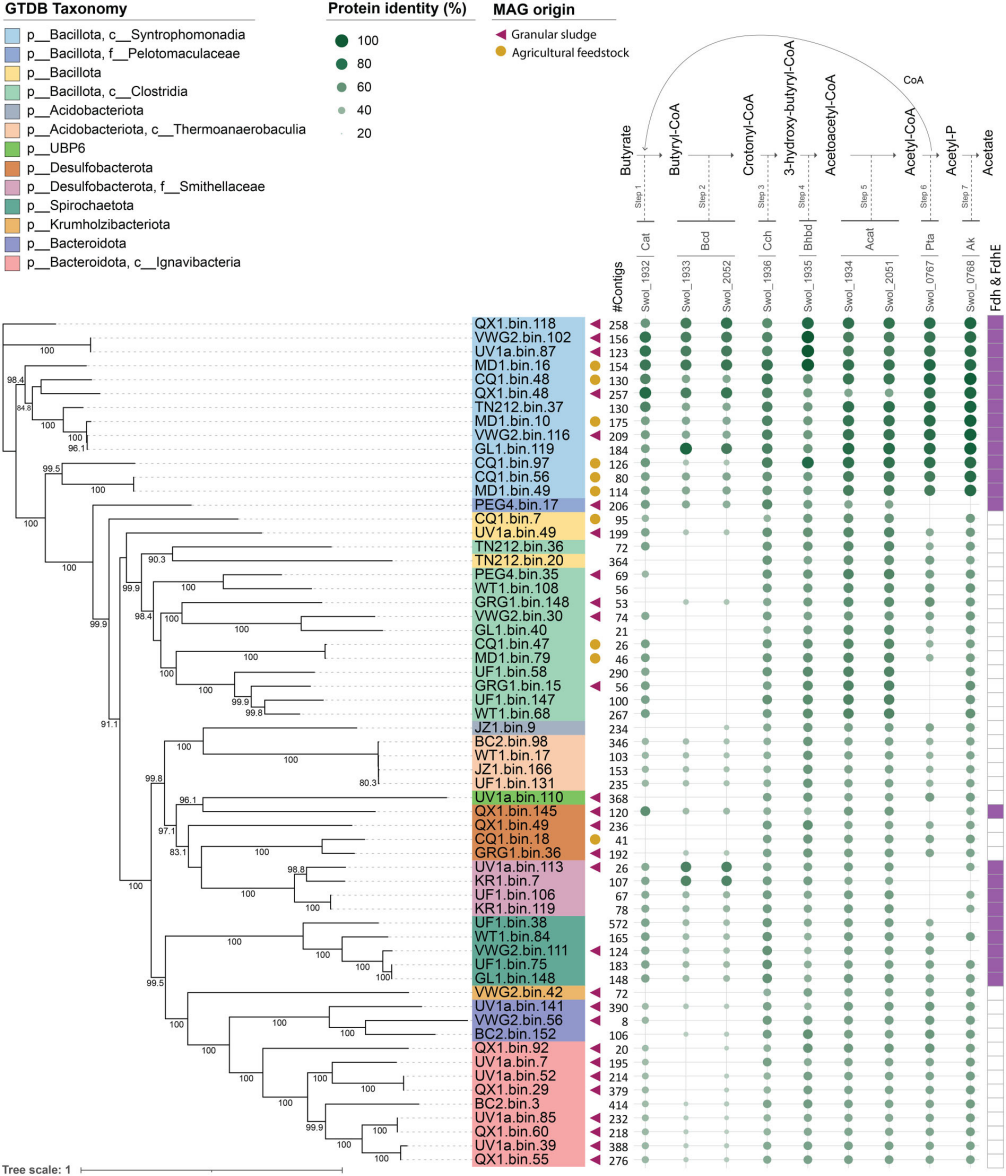
HC-MAGs containing homologs for the syntrophic butyrate oxidation pathway of *Syntrophomonas wolfei*. All HC-MAGs included contain a homologs for at least five steps of the pathway. HC-MAGs not originating from granular sludge or from sludge fed with agricultural waste originated from sludge processing municipal wastewater. The phylogenetic tree is unrooted and is based on a trimmed alignment of 120 bacterial marker genes using GTDB-Tk and is constructed with substitution model LG+R6 (Nuclear region with general matrix and FreeRate model) and 1000 non-parametric fast bootstrap replicates. Bootstrap values >80 are displayed. Purple boxes represent presence of Fdh and FdhE. Abbreviations: Cat, butyrate: acetyl-CoA coenzymeA-transferase; Bcd, butyryl-crotonyl dehydrogenase; Cch, crotonyl-CoA hydratase; BHBD, 3-hydroxybutyryl-CoA dehydrogenase; Acat, acetyl-CoAacetyl transferase; Pta, phosphate acetyltransferase; Ak, acetate kinase.

Of the 743 assembled HC-MAGs, MAGs containing homologues for genes involved in β-oxidation of butyrate (based on *S. wolfei*) are shown in Fig. 4. Bacillota MAGs of the class Syntrophomonadia were identified, known to include butyrate oxidizing syntrophs. Surprisingly, most were recovered from either granular sludge or sludge fed with agricultural waste. While most MAGs of this class belong to the family Syntrophomonadaceae, two other and yet uncultivated families are identified: CALXSZ01 (UV1a.bin.87 and VWG2.bin.102) and DTU052 (MD1.bin.49 and CQ1.bin.56). All MAGs of the class Syntrophomonadia contain homologs for the six selected SGA protein domains, except for MD1.bin.16 missing the RNAseP homolog (Fig. 4 and File S1, “Butyrate”). All other MAGs within the phylum Bacillota do not contain homologs for the complete butyrate oxidation pathway, mostly missing either the butyrate: acetyl-CoA coenzymeA-transferase (Cat), butyryl-CoA dehydrogenase (Bcd), or phosphate-transacetylase (Pta).

Besides the phylum Bacillota, three other phyla contain MAGs with homologs for a complete butyrate oxidation pathway: four MAGs of the phylum Acidobacteriota, three MAGs of the phylum Spirochaetota, and nine MAGs of the phylum Bacteroidota. None of the Acidobacteriota MAGs contain homologs for both the Fdh and the FdhE SGA protein domains. The complete formate dehydrogenase (Fdh) protein plays a key role in reverse electron transfer in syntrophic organisms, coupling oxidation of NADH to reduction of CO_2_ to formate. This is an essential mechanism for syntrophs to conserve energy on the thermodynamic limit of life. All Spirochaetota MAGs are classified in the uncharacterized class UBA4802 and most contain all SGA protein domains, with only one MAG missing the SGA protein domain RNAseP (File S1, “Butyrate”), possibly due to MAG incompleteness. Of particular interest are nine MAGs in the phylum Bacteroidota classified as Ignavibacteria, with only one MAG missing a Bcd required for butyryl oxidation. Besides MAG BC2.bin.3, all MAGs classified as Ignavibacteria originate from Granular sludge samples. One other Bacteroidota MAG, UV1a.bin141, further classified as the uncharacterized family TTA-H9 (genus GWE2-42-24), contains homologs for the complete pathway. All Bacteroidota MAGs however lack multiple homologs of the selected SGA protein domains, including both the Fdh and FdhE proteins.

For all other MAGs, homologs for up to two steps of the butyrate oxidation pathway were missing. Noteworthy are four Desulfobacterota MAGs belonging to the family Smithellaceae, which is known to oxidize propionate into acetate and butyrate. These MAGs contain homologs for all proteins except for Pta, which recycles Coenzyme A by replacing it with a phosphate group. Of all SGA protein domains, the Smithellaceae MAGs only miss either the CapA or the RNaseP SGA (File S1, “Butyrate”). The other four Desulfobacterota MAGs, all originating from granular sludge or sludge fed with agricultural waste, belong to the classes Desulfomonilia, Desulfobulbia, or Deferrimicrobia and miss either the Cat, Bcd, or Pta of the pathway (Fig. 4). Of these four MAGs, the Desulfomonilia MAG (QX1.bin.145) is the only one containing a homolog for all SGA protein domains.

### Exploring syntrophic propionate oxidation in AD MAGs

**Figure 5:**
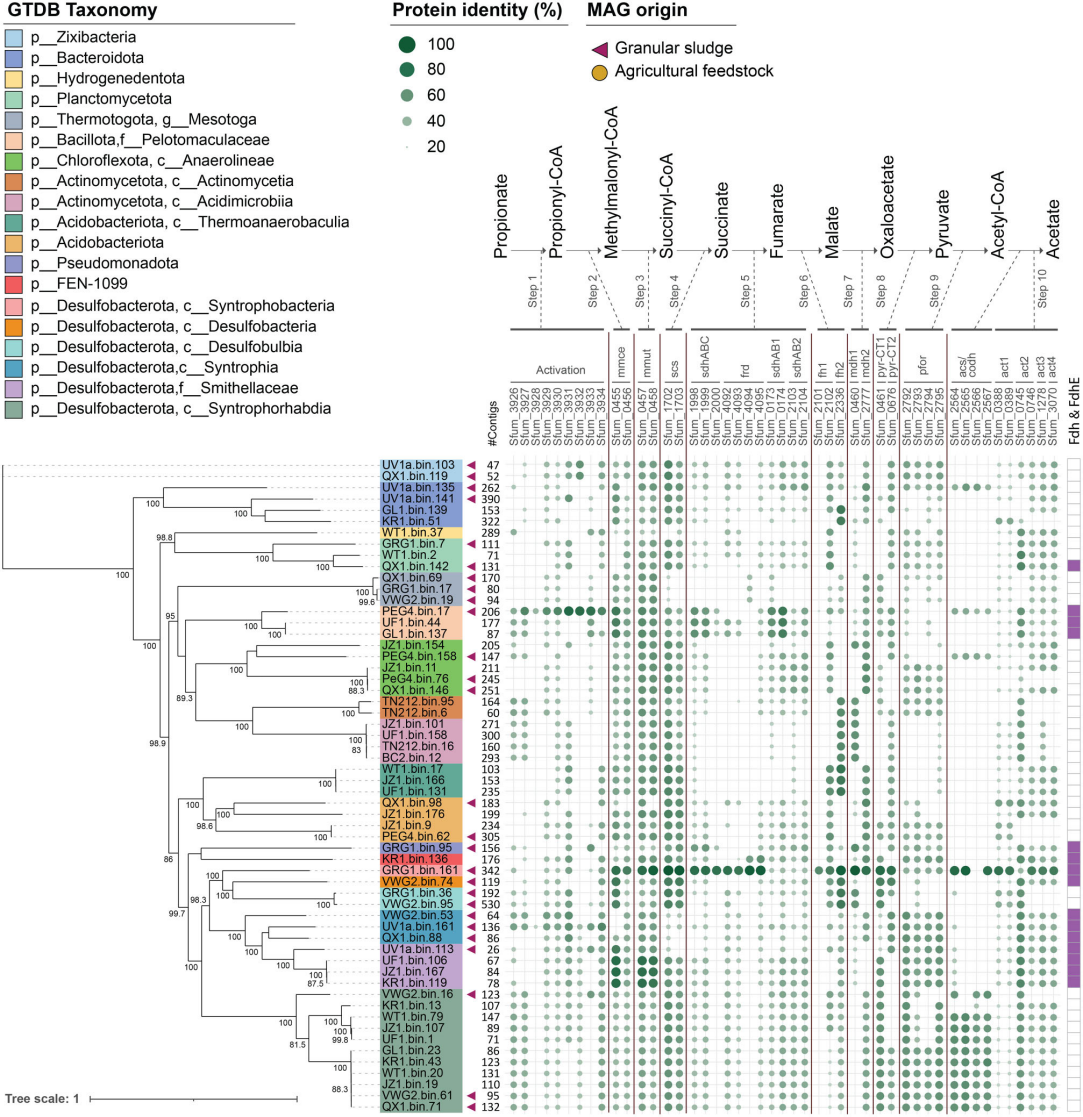
MAGs containing homologs for the syntrophic propionate oxidation pathway of *Syntrophobacter fumaroxidans*. All MAGs included contain at least one homolog for each of the 10 steps. The phylogenetic tree is unrooted and is based on a trimmed alignment of 120 bacterial marker genes using GTDB-Tk and is constructed with substitution model LG+I+F+R5 (Nuclear region with general matrix, allowing for a proportion of invariable sites, empirical base frequencies plus FreeRate model) and 1000 non-parametric fast bootstrap replicates. Bootstrap values >80 are displayed. Purple boxes represent presence of Fdh and FdhE. Abbreviations: Mmce, methylmalonyl-CoA epimerase; mmut, methylmalonyl-CoA mutase; scs, succinyl-CoA synthetase; sdh, succinate dehydrogenase; frd, fumarate reductase; fh, fumarase/fumarate hydratase; mdh, malate dehydrogenase; pyr-CT, pyruvate carboxyl transferase; ACS/CODH, acetyl-CoA synthase/carbon monoxide dehydrogenase; PFOR, pyruvate: ferredoxin oxidoreductase; act, acetyl-CoA transferase.

Of the 743 assembled HC-MAGs, MAGs containing homologues for genes involved in the MMC pathway for propionate oxidation (based on *S. fumaroxidans*) are shown in Fig. 5. MAGs of the three propionate-oxidizing families Pelotomaculaceae (phylum Bacillota) and Smithellaceae and Syntrophobacteraceae (phylum Desulfobacterota) that could be expected to be found were identified. Only one of these MAGs is classified as a known SPOB: *P. propionicum* (PEG4.bin.17). Novel potential SPOB identified within these families were the Syntrophobacteraceae species JAAYUJ01 sp012517775, the Smithellaceae species UBA4810 sp002418825 and genus UBA8904, and the Pelotomaculaceae species DTU098 sp002305915 (File S1, “Propionate”). Interestingly, the identified *P. propionicium* is the only MAG with complete propionate activation homologs in high sequence similarity to the query operon.

Focusing on the pathway from propionyl-CoA onward, complete pathways were only identified in the phylum Desulfobacterota. Of particular interest is the cluster of MAGs of the family Syntrophorhabdia, with six MAGs having a complete pathway from propionyl-CoA onward. The other five Syntrophorhabdia MAGs miss complete ACS/CODH and/or PFOR operons. SGA protein domain FdhE was not detected, but 10 out of 11 Syntrophorhabdia MAGs do contain a Fdh (File S1, “Propionate”). Two MAGs missing only the ACS/CODH cluster close to the phylum Desulfobacterota: one MAG of the phylum Pseudomonadota and one MAG of the *Candidate* phylum FEN-1099. FEN-1099, proposed *Candidatus* Lernaellota or *Candidatus* Podoxiota (Pallen et al. 2022, Williams et al. 2022), contains homologs for all SGA protein domains. The Pseudomonadota MAG, classified as *Thauera* sp003963015, belongs to the family Rhodocyclaceae and misses only the CapA and RNaseP homologs. One MAG of the phylum Actinomycetota, class Acidimicrobia, also has homologs for both Fdh and FdhE proteins. However, it has no complete sdh, PFOR, or ACS/CODH operon. Even though these organisms miss genes from the MMC pathway, they all exhibit potential for production of the SGA protein domain for the extra-cytoplasmic protein Fdh and for multiple other SGA protein domains.

It stands out that of all selected MAGs, no MAGs originating from sludge fed on agricultural waste had genomic potential for propionate oxidation according to the set thresholds. This suggests propionate might play a less prominent role in this type of waste conversion. Furthermore, 43% of all selected MAGs originate from granular samples. As granules are in general less well studied and less used in cultivation attempts compared to suspended sludges, the potentially syntrophic MAGs identified are an especially interesting starting point for characterization of novel syntrophic species. All MAGs classified as Syntrophobacteria, Desulfobacteria, Desulfobulbia, and Syntrophia originate from granular sludge samples. This includes the three MAGs of class Syntrophia that belong to uncharacterized families UBA6807, PHBD01, and FEN-1087, closest related to the known SPOB family Smithellaceae.

### Exploring syntrophic acetate oxidation in AD MAGs

**Figure 6:**
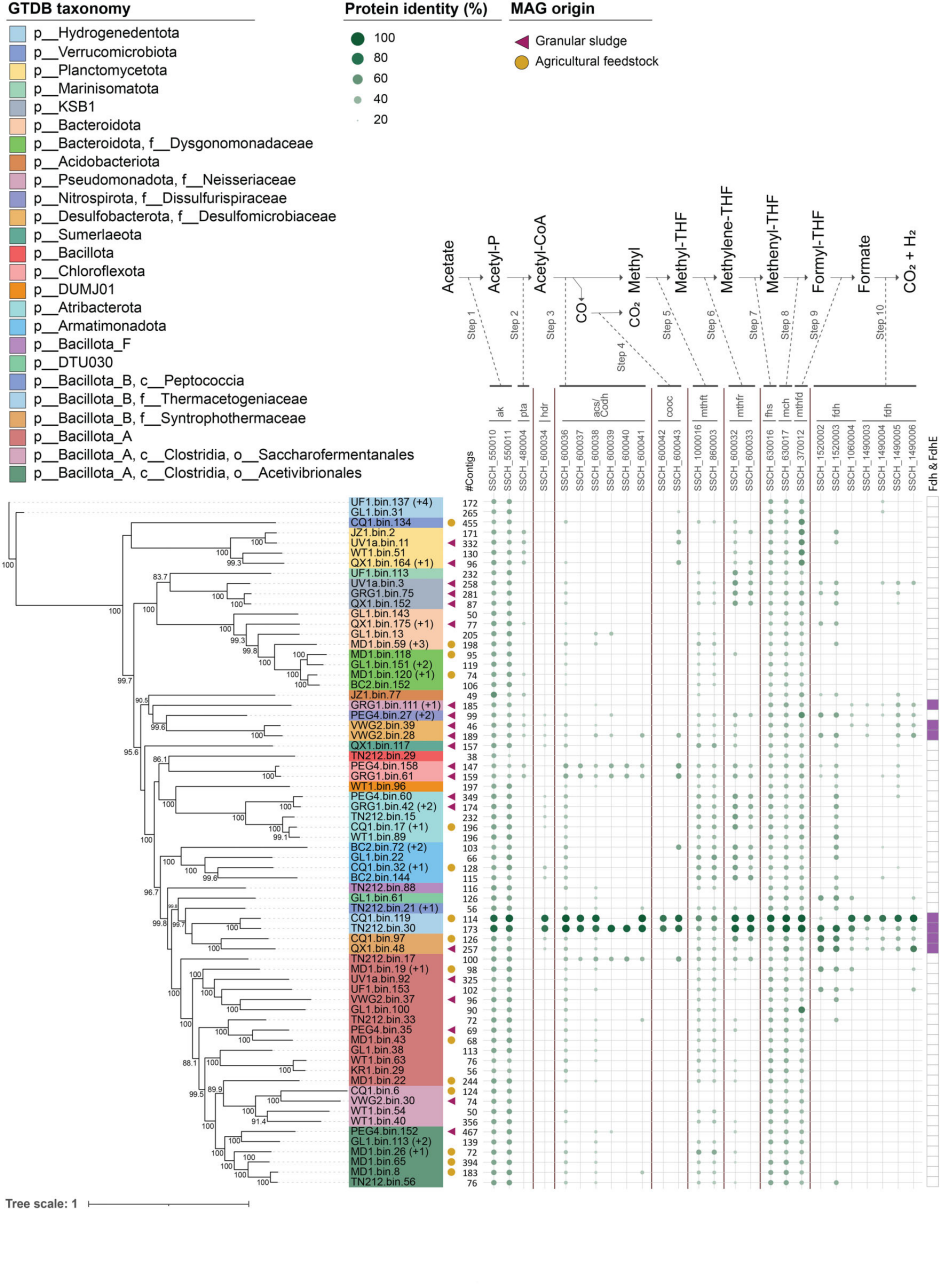
MAGs containing homologs for the syntrophic acetate oxidation pathway of *Syntrophaceticus schinkii*. All MAGs included contain at least homologs for acetate activation and for conversion from methylene-THF to formate. The phylogenetic tree is unrooted and based on a trimmed alignment of 120 bacterial marker genes using GTDB-Tk and is constructed with substitution model LG+I+R7 (Nuclear region with general matrix, allowing for a proportion of invariable sites plus FreeRate model) and 1000 non-parametric fast bootstrap replicates. Bootstrap values >80 are displayed. Purple boxes represent presence of Fdh and FdhE. Of MAG clusters sharing the same phylogeny and having a similar heatmap pattern, only the most complete MAG is included in this figure. Homolog data of all MAGs can be found in File S1 (tab “Acetate”). Abbreviations: ak, acetate kinase; Pta, phosphotransacetylase; hdr, heterodisulfide reductase; acd/CODH, acetyl-CoA synthase/Carbon monoxide dehydrogenase; mtfht, Methyl-THF transferase; mthfr, methylene THF reductase; fhs, formyl THF synthetase; mch, methenyl THF cyclohydrolase; mthfd, methylene tetrahydrofolate (THF) dehydrogenase; fdh, formate dehydrogenase.

Of the 743 assembled HC-MAGs, MAGs containing homologues for genes involved in the reverse WLP for acetate oxidation (based on *S. schinkii*) are shown in Fig. 6. With many uncertainties in the acetate oxidation pathway of known syntrophic acetate oxidizers, only MAGs were selected with homologs (>20% protein similarity) to the genes that are found in multiple verified syntrophic acetate oxidizers. Therefore, MAGs are included if they contain homologs for the acetate kinase and for the genes involved in conversion of methylene-THF to formate (Fig. 6, step 1 and steps 7–9) (Manzoor et al. 2018). Of the 98 selected MAGs, 26 duplicate MAGs were removed for visualization (see the section “Methods”).

Of all identified MAGs with potential for SAO, only two MAGs classified as the family Thermacetogeniaceae were found to have an almost complete pathway of *S. schinkii*, classified as genera *Syntrophaceticus* and DTU068. They contain high similarity homologs, but no phosphotransacetylase (Pta) and only one ACS/CODH complex was identified. The two MAGs clustering closest to the Thermacetogeniaceae MAGs are classified in the family Syntrophothermaceae, genera UBA5314 and JAAZLJ01. All other MAGs miss many protein homologs with on average 60% of the pathway lacking. They can be considered potential organisms of interest as they both also have homologs for all SGA protein domains (File S1, “Acetate”).

Both Thermacetogeniaceae MAGs and Syntrophothermaceae MAGs belong to the phylum Bacillota, the biggest represented phyla in the search for this pathway of *S. schinkii* with 35 selected MAGs. While in no other Bacillota family the protein domains for the Fdh and FdhE were identified, most do contain homologs for CapA, RNaseP and cell cycle proteins FtsW, RodA, and SpoVE. Outside of the Bacillota phylum, only two MAGs of the phylum Chloroflexota family 4572–78, both originating from granular sludge, seem to have a near-complete pathway, but they lack complete operons for either the soluble or membrane-bound formate dehydrogenases and miss most of the SGA protein domains. Considering the limited knowledge available on SAO pathways, the lack of a complete WLP identified in the high number of Bacillota MAGs cannot exclude a potential role in syntrophic acetate oxidation.

Outside of the Bacillota phylum, three MAGs are identified with both Fdh and FdhE SGA protein domains. One MAG of the genus *Neisseria* (phylum Pseudomonadota) and two MAGs of the genus *Desulfomicrobium* of which one further classified as species sp023393525 (phylum Desulfobacterota). All three originate from granular sludge and are phylogenetically placed closely to other MAGs of granular sludges, including a MAG of the recently discovered family Dissulfurispiraceae (Umezawa et al. 2021), classified as JAAYUK01 sp012517735. While no complete WLP could be identified in these MAGs, this group of MAGs of granular sludge origin pose an interesting observation in the search for potentially novel SAOB.

## Discussion

This study set out to provide insight on the presence of syntrophs in suspended and granular sludges from anaerobic digestion systems across the Netherlands, focusing on potential novel syntrophs. A comprehensive screening of 111 AD samples by 16S rRNA gene amplicon sequencing showed a high relative abundance of already known syntrophic groups in granular sludges, leading to question whether more novel groups could be identified in these systems. While specific discrimination between suspended and granular sludges is often missing in other studies, here, multiple HC-MAGs of yet uncharacterized and uncultivated phyla were identified from granular sludge samples. Differences in feedstocks between suspended and granular sludges of this study should be recognized, but the high number of MAGs originating from granular sludges and assigned to yet uncharacterized phyla does show that granular sludges are a good starting point when focusing on identification and subsequent cultivation of novel microbial groups.

### Identified known syntrophs

The search for the genomic potential of syntrophic degradation of butyrate, propionate and acetate expectedly led to identification of known and verified syntrophic groups. These included potential butyrate oxidizers within the class Syntrophomonadia, propionate oxidizers within families Syntrophobacteraceae, Pelotomaculaceae, and Smithellaceae, and acetate oxidizers within the family Thermacetogeniaceae. Surprisingly, Smithellaceae-related MAGs were identified in both searches for butyrate and for propionate oxidation pathways. Previous studies have identified *Smithella* sp. as SPOB, which is supported by various cultivation attempts (Jannat et al. 2021, Liu et al. 1999). *Smithella* spp. are however not proposed to oxidize propionate via the MMC pathway searched for in this study, but via a dismutation pathway into butyrate and acetate (De Bok et al. 2001). The proteins involved in the dismutation pathway have however not yet been identified. The homologs identified in this study present an interesting starting point to further study the route of propionate oxidation that *Smithella* spp. employ.

Syntrophic butyrate oxidation is not commonly identified outside of the class Syntrophomonadia, but has been shown for four organisms classified within the Desulfobacterota phylum: two *Candidatus* Phosphitivorax spp., and two MAGs classified in the class Desulfomonilia (Hao et al. 2020). In the current study, two groups previously identified as syntrophic LCFA degraders (Sun et al. 2022) were also identified as potential syntrophic butyrate degraders: one MAG classified in the class Desulfomonilia sp. (family UBA1062) and five MAGs in the phylum Spirochaetota (families UBA5368 and UBA5550). With also all SGA protein domains identified in these groups, this data shows the possibility of more syntrophic butyrate degraders outside of the class Syntrophomonadia.

Another group of confirmed syntrophs that unexpectedly showed-up is the class Syntrophorhabdia. The only described member of the class Syntrophorhabdia is *Syntrophorhabdus aromaticivorans* strain UI, which was isolated in 2008 in a coculture with *Methanospirillum hungatei* degrading the aromatic compound phenol (Qiu et al. 2008, Qiu et al. 2004). Strain UI is unable to grow on propionate as carbon source, along with 87 other tested substrates including intermediates of the MMC pathway succinate, malate and pyruvate (Qiu et al. 2008). In this study, however, eleven Syntrophorhabdia MAGs showed potential for syntrophic propionate degradation (genera *Syntrophorhabdus*, MWEV01, and Delta-02). Cultivation of more organisms within the class Syntrophorhabdia is essential to confirm the possibility of propionate oxidation for other species within the genus *Syntrophorhabdus*, but so far this family has only been linked to syntrophic degradation of aromatic compounds (Kuroda et al. 2022, Nobu et al. 2017, Kuroda et al. 2024, Junghare et al. 2019). This is however also the case for the only two other isolated syntrophic aromatic compound oxidizers *P. isophthalicum* strain JI and *P. terephthalicum* strain JT (Qiu et al. 2006). They also were not able to grow on propionate—or on pathway intermediates, such as succinate, malate or pyruvate—but they do classify within the genus *Pelotomaculum*, known to contain propionate oxidizing syntrophs (Imachi et al. 2002, 2007).

The search for potential novel acetate oxidizers mainly highlighted the ambiguity around known pathways of syntrophic acetate oxidation. This study was limited to the only verified pathway for syntrophic acetate oxidation pathway (i.e. the reverse WLP) used by *S. schinkii*. The poorly understood mechanisms of SAOB become clear from the lack of homologs found for the pathway. Only two MAGs of the family Thermacetogeniaceae contained a complete pathway, one of the same genera as *S. schinkii—Syntrophaceticus*—and one representing a novel genus: DTU068. The close phylogenetic association of DTU068 to genus *Syntrophaceticus* shows the potential for this MAGs to represent a novel SAO. Closely affiliated to the Thermacetogeniaceae MAGs were two Syntrophothermaceae MAGs. Interestingly, these have previously been suggested to be involved in syntrophic oxidation of butyrate or propionate, not acetate (Yan et al. 2020).

### Potential new syntrophic microorganisms

The identification of multiple syntrophic groups emphasizes the potential that homolog searches have for identification of new interesting targets from large data sets. The lack of information on pathway direction however remains a limiting factor when using a genomic approach. The hereafter highlighted organisms should therefore be considered solely to have the indication of being potentially syntrophic (Table 3), for which further research efforts, including cultivation approaches, remain essential to determine their full metabolic potential.

**Table 3:** Overview of potential syntrophic candidates identified by metagenomic analysis searching for genomic capacity of oxidizing volatile fatty acids butyrate, propionate, or acetate.

	Taxonomy	Examples of other MAGs isolation sources
**Butyrate**	p__Bacillota_B; c__Syntrophomonadia; **o__Syntrophomonadales; f__CALXSZ01**	Chicken caecal content
	p__Bacillota_B; c__Syntrophomonadia; **o__Syntrophomonadales; s__DTU052 sp001512495**	Thermophilic AD sludge treating cattle manure
	p__Spirochaetota; c__UBA4802; o__UBA4802; **f__UBA5368; g__MVZN01; s__MVZN01 sp002069125**	AD sludge
	p__Spirochaetota; c__UBA4802; o__UBA4802; **f__UBA5550; g__UBA5550 / g__JAAYBQ01**	AD sludge, wastewater
	p__Bacteroidota; c__Ignavibacteria; **o**__Ignavibacteriales; **f__Melioribacteraceae; g__JAEXOD01**	No other available genomes
	p__Bacteroidota; c__Ignavibacteria; **o**__Ignavibacteriales; **f__Ignavibacteriaceae; s__UTCHB3 sp016707285**	Activated sludge, wastewater, Biofilm anammox reactor
	p__Bacteroidota; c__Ignavibacteria; **o**__Ignavibacteriales; **f__Ignavibacteriaceae; g__JAAYVN01**	AD sludge, Biofilm anammox reactor
**Propionate**	p__Desulfobacterota; c__Syntrophobacteria; o__Syntrophobacterale; **f__Syntrophobacteraceae; s__JAAYUJ01 sp012517775**	AD sludge
	p__Desulfobacterota; c__Syntrophia; o__Syntrophales; **f__Smithellaceae; s__UBA4810 sp002418825**	AD sludge, wastewater,
	p__Desulfobacterota; c__Syntrophia; o__Syntrophales; **f__Smithellaceae; g__UBA8904**	AD sludge, wetland soil, wastewater, sediment, leachate well, groundwater, lake water
	p__Bacillota_B; c__Desulfotomaculia; o__Desulfotomaculales; **f__Pelotomaculaceae; s__DTU098 sp002305915**	AD sludge, biodigester
	p__Desulfobacterota_G; c__Syntrophorhabdia; o__Syntrophorhabdales; **f__Syntrophorhabdaceae; s__MWEV01 sp002071245**	AD sludge, wastewater
	p__Desulfobacterota_G; c__Syntrophorhabdia; o__Syntrophorhabdales; **f__Syntrophorhabdaceae; s__Syntrophorhabdus sp002067585**	AD sludge, wastewater, methanogenic bioreactor treating terephthalic acid process wastewater,
	p__Pseudomonadota; c__Gammaproteobacteria; o__Burkholderiales; **f__Rhodocyclaceae; s__Thauera sp003963015**	Wastewater
	p__FEN-1099; c__FEN-1099; o__FEN-1099; **f__FEN-1099;** g__JAAYVF01	AD sludge Note: proposed phylum name *Ca*. Lernaellota or *Ca*. Podoxiota
	p__Desulfobacterota; c__Syntrophia; **o__Syntrophales; f__UBA6807**; g__UBA6807	Wastewater, AD sludge
	p__Desulfobacterota; c__Syntrophia; **o__Syntrophales; f__PHBD01**; g__PHBD01	Groundwater, 2.8-km deep subsurface aquifer
	p__Desulfobacterota; c__Syntrophia; **o__Syntrophales; f__FEN-1087**; g__FEN-1087	Permafrost active layer soil
**Acetate**	p__Bacillota_B; c__DSM-12270; o__Thermacetogeniales; **f__Thermacetogeniaceae; s__DTU068 sp001513545**	Thermophilic AD sludge treating cattle manure, AD sludge
	p__Bacillota_B; c__Syntrophomonadia; o__Syntrophomonadales; **f__Syntrophomonadaceae; g__UBA5314**	Mud, AD sludge
	p__Bacillota_B; c__Syntrophomonadia; o__Syntrophomonadales; **f__Syntrophothermaceae; s__JAAZLJ01 sp012799365**	AD sludge
	p__Chloroflexota; **c__Anaerolineae; o__4572–78**; f__4572–78	Deep-sea hydrothermal vent field and sediments

Addressing the main goal of this study, we found several groups potentially containing new potential syntrophic microorganisms. The first search for potential novel butyrate oxidizers highlighted two new families within the class Syntrophomonadia: DTU052 and CALXsZ01. *Syntrophothermus* sp. DTU052 was identified in sludge fed with agricultural waste and was previously identified in a lab-scale biogas upgrading reactor linked to beta-oxidation activity (Treu et al. 2018). In that study, the sampled biogas upscaling reactor was fed with digestate of a primary reactor, which included livestock manure in the reactors feedstock, similar to the feedstock in our reactor systems. Family CALXSZ01, on the other hand, was only identified in granular sludge samples, but no reports on its potential syntrophic butyrate degrading capacity existed at the time of writing. Being very closely related to butyrate-oxidizing family Syntrophomonadaceae does infer that syntrophic butyrate oxidation is likely for family CALXSZ01, potentially as a more dominant group in granular AD sludges.

The search for potential novel propionate oxidizers highlighted multiple novel and uncharacterized groups of interest: Syntrophia families UBA6807, PHBD01, and FEN-1087. They were all recovered from granular sludges and closely related to the family Smithellaceae. FEN-1087 was previously mentioned to belong to Deltaproteobacteria subgroup G and is herein proposed to be likely involved in syntrophic interactions based on the genomic phylogenetic placement and limited metabolic abilities, such as absence of a sulfate reductase (Langwig et al. 2022). No reports have been found on the metabolic capabilities of Syntrophia families UBA6807 and PHBD01, but their close phylogenetic affiliation to FEN-1087 and *Smithella* spp. suggests a similar metabolic role. The MAG of family PHBD01, UV1a.bin.161, clusters closest to family FEN-1087. Interestingly, this MAG is the only one besides the Pelotomaculaceae MAG with homologs to all proteins of *S. fumaroxidans* potentially involved in propionate oxidation, including propionate activation genes. Further efforts to elucidate the propionate oxidation pathway of *Smithella* spp. is essential to uncover more potential SPOB in addition to investigating the potential novel syntrophic propionate oxidizing families UBA6807, PHBD01, and FEN-1087.

Two final groups with a potential for syntrophic propionate degradation were within the phylum FEN-1099, proposed Candidatus *Lernaellota* (also referred to as Candidatus *Podoxiota* (Pallen et al. 2022, Williams et al. 2022)), and *Thauera* sp003963015 of the family Rhodocyclaceae. Candidatus *Lernaellota* has been suggested to be involved in nitrogen cycling, or degradation of polymeric organic matter (Qiu et al. 2024, Williams et al. 2022). Organisms belonging to the genus *Thauera* are generally known as denitrifiers and are commonly found in wastewater systems (Chen et al. 2016, Guo et al. 2019). For neither group a link to potential syntrophic growth has been made in previous studies. The presence of all SGA protein domains in the MAG of Candidatus *Lernaellota* does suggest that a syntrophic metabolism should not be excluded yet.

Besides the four known syntrophic Bacillota MAGs identified in the search for novel syntrophic acetate oxidation, there were only three MAGs that stood out with both Fdh and FdhE SGA protein domains present: one MAG of the genus Neisseria (phylum Pseudomonadota) and two MAGs of the genus *Desulfomicrobium* of which one further classified as species sp023393525 (phylum Desulfobacterota). *Desulfomicrobium* belongs to the order Desulfovibrionales, members of which are known to grow both syntrophically and non-syntrophically with sulfate as electron acceptor (Badziong et al. 1978, Plugge et al. 2010). It is therefore more likely that the MAGs identified here are not strictly syntrophic organisms. The genus *Neisseria* is associated with two pathogenic species, *Neisseria gonorrhoeae* and *Neisseria meningitidis* (Walsh et al. 2023), which are unlikely to grow as syntrophic acetate oxidizers.

Intriguing is the MAG TN212.bin.17, classified as *Tepidanaerobacter* sp. DTU063, which has previously been proposed as a potential novel syntrophic acetate oxidizer (Treu et al. 2018). This MAG has homologs for a complete ACS/CODH, which were only identified in four other genomes: the two Thermacetogeniaceae MAGs and two MAGs classified as Chloroflexota, class Anaerolineae and family 4572–78. Members of the phylum Chloroflexota are often found in AD systems and are predicted to play an essential role in syntrophic methanogenic communities (McIlroy et al. 2017, Petriglieri et al. 2018). In a recent study, the isotopic labeling of acetate in lab-scale chemostats also showed the possibility of Anaerolineae being involved in acetate oxidation (Zheng et al. 2019). Additionally, two *Dehalococcoides* sp. isolates have been shown to use acetate as carbon source, but in these examples the organisms were not grown syntrophically but with either chlorinated ethene or trichlorobenzene as electron acceptors (Löffler et al. 2013).

## Conclusions and future directions

Metagenomes represent a useful source for identification of novel and potentially important lineages within, for example, full industrial AD reactor systems. Even though metagenomic analyses are limited to only reveal potential metabolic pathways without possibility to conclude on actual functions within a community as a whole, they form a basis for follow-up studies focusing in detail on novel identified lineages and their potential role and interdependencies within a system. This is evident from the exploratory nature of this study where still multiple new families and genera were identified that are worth of further investigation regarding their potential as syntrophic fatty acid oxidizers. MAGs with incomplete pathways and/or low-identity homologs, as were found for multiple novel groups, should not be excluded for future research. First, because those MAGs are incomplete and the missing genes can still be present in the part of the genome that was not recovered. Second, homologs with lower similarity likely represent genes that are more distantly related to the genes of the known syntrophic taxa. Last, it is possible that a gene performing a similar function is simply not yet identified. A high fraction of homologs identified in a pathway could still infer a potential role in AD involving syntrophic VFA oxidation, especially for MAGs in which multiple SGA protein domains were identified.

In this study, we also increased the knowledge of potentially important lineages present in granular AD sludges. The microbial diversity present in anaerobic microbial granules deserves more focus in future studies as many groups identified here from granular sludges are not yet cultivated. Cultivation approaches can focus on physical separation between individual cells and their environments, *e.g*. via membrane-based techniques or via cell-sorting, or they can make use of genomic data and design targeted methods for isolation (Lewis et al. 2021). Using reverse genomics, antibodies for membrane-associated proteins targeting individual microbial groups can be designed (Cross et al. 2019). This technique will increase the chance for isolation of novel organisms, allowing physiological characterization. Developments in automatization of the antibody selection process are needed to establish a more time-feasible approach. The high completion of the MAGs obtained in this study and the selected targets provide a good starting point for future studies focusing on reverse genomics or other targeted cultivation for isolation of novel species.

## Supplementary Material

fiag052_Supplemental_File

## Data Availability

Raw sequencing data and the assembled MAGs were deposited to the European Nucleotide Archive at project PRJEB87655. Supplementary material File S1 has been deposited to the Zenodo EU Open Research Repository with accession number 10.5281/zenodo.15261234.
